# Bioremediation of Hazardous Pollutants Using Enzyme-Immobilized Reactors

**DOI:** 10.3390/molecules29092021

**Published:** 2024-04-27

**Authors:** Hiroshi Yamaguchi, Masaya Miyazaki

**Affiliations:** 1 Department of Food and Life Science, School of Agriculture, Tokai University, 871-12 Sugido, Mashiki, Kamimashiki, Kumamoto 861-2205, Japan; 2 Graduate School of Agriculture, Tokai University, 871-12 Sugido, Mashiki, Kamimashiki, Kumamoto 861-2205, Japan; 3 Graduate School of Bioscience, Tokai University, 871-12 Sugido, Mashiki, Kamimashiki, Kumamoto 861-2205, Japan; 4 HaKaL Inc., Kurume Research Park, 1488-4 Aikawa, Kurume, Fukuoka 839-0864, Japan; miyazaki@hakal-jp.com

**Keywords:** bioremediation, immobilized enzyme, bioreactor, pollutant, degradation

## Abstract

Bioremediation uses the degradation abilities of microorganisms and other organisms to remove harmful pollutants that pollute the natural environment, helping return it to a natural state that is free of harmful substances. Organism-derived enzymes can degrade and eliminate a variety of pollutants and transform them into non-toxic forms; as such, they are expected to be used in bioremediation. However, since enzymes are proteins, the low operational stability and catalytic efficiency of free enzyme-based degradation systems need improvement. Enzyme immobilization methods are often used to overcome these challenges. Several enzyme immobilization methods have been applied to improve operational stability and reduce remediation costs. Herein, we review recent advancements in immobilized enzymes for bioremediation and summarize the methods for preparing immobilized enzymes for use as catalysts and in pollutant degradation systems. Additionally, the advantages, limitations, and future perspectives of immobilized enzymes in bioremediation are discussed.

## 1. Introduction

Environmental pollution, which has been caused by population growth and industrial development, represents a serious worldwide problem [1]. Water is indispensable, but securing stable supplies of clean water has become a global issue [2]. Recently, various hazardous pollutants, such as pesticides, dyes, heavy metals, pharmaceuticals, plastics, and other chemicals released by personal products, have been outflowing into the environment at increasing rates, and their negative impacts on other living organisms pose problems even at low concentrations [3,4]. These hazardous pollutants reportedly have serious effects on human health [5,6], including growth inhibition, promotion of antibiotic resistance, behavioral changes, reproductive impairments, and endocrine system disruption. Therefore, these pollutants must be removed or degraded before they are discharged into the environment. Accordingly, technologies to purify industrial and domestic wastewater have advanced. Wastewater is typically treated by physical and chemical methods. Physical methods, such as coagulation, sedimentation, and filtration, involve the removal of pollutants from wastewater without altering their biochemical properties. Although this method is simple, it requires human resources and energy. Chemical methods use compounds such as oxidizers to remove pollutants via chemical reactions [7,8]. Industrial and agricultural waste can be effectively treated by chemical methods; however, the addition of large amounts of chemical compounds to agricultural fields can affect the quality of the soil, enter agricultural water, and cause cross-contamination. Consequently, applying these physical and chemical methods poses various challenges, such as the generation of byproducts and secondary wastes, the use of toxic chemical compounds, high costs, and the need for sophisticated equipment [9]. Therefore, it is important to develop new removal and degradation methods that are eco-friendly, sustainable, and minimize waste.

Bioremediation uses the degradative ability of microorganisms and other organisms to remove harmful pollutants from the natural environment and return them to a pollutant-free state [10,11,12,13]. The advantage of using natural microorganisms for wastewater treatment is that they have a lower environmental impact than physical and chemical methods. However, harmful pollutants can be toxic to degrading microorganisms at high concentrations. Recently, both microorganisms and enzymes derived from them have been shown to efficiently degrade and eliminate a variety of pollutants, transforming them into non-toxic forms [9,14,15,16]. In contrast to using microorganisms, using enzymes has many advantages, such as minimal waste production, low toxicity, and the capability to operate under mild conditions. However, enzymes, which are proteins, are often difficult to use in large-scale industries owing to their instability and restricted reusability [17]. Immobilized enzymes are widely used to improve operational stability, catalytic properties, and efficiency [18,19,20,21]. Immobilized enzymes have several advantages over free enzymes [20,21,22,23]. The enzyme can be immobilized on a support material at high concentrations, leading to a high catalyst concentration in the reaction system. Moreover, the stability and reusability of immobilized enzymes are reportedly higher than those of free enzymes [21,24,25]. Therefore, immobilized enzymes provide heterogeneous catalysts with enhanced stability under various reaction conditions, reusability, and overall cost minimization [26]. The immobilization method depends on the type of enzyme, support material, intended application, and operating conditions [27]. Therefore, selecting suitable support materials and immobilization methods is essential for efficient bioremediation.

In recent years, enzyme-immobilized reactors have been used to degrade pollutants and are expected to become commercially available in the future. Although several reviews have been reported to develop and evaluate enzyme-immobilized reactors for the bioremediation of hazardous pollutants, this is the latest review of recent advances, advantages, limitations, and future perspectives of enzyme-immobilized reactors in the last five years. Enzyme immobilization techniques suitable for bioremediation are also highlighted. In addition, the applications, limitations, and future perspectives of enzyme-immobilized reactors for the degradation of hazardous pollutants, including dyes, phenolic compounds, and pharmaceuticals, are discussed. 

## 2. Immobilization Methods for Enzyme-Immobilized Reactors

Enzymes have high catalytic activity, substrate specificity, and the ability to degrade pollutants. Immobilizing enzymes on support materials without losing their catalytic activity requires their conformational structures to remain intact. Microspheres, beads, and hydrogels are widely used as support materials because of their durability, biocompatibility, stability, inactivity, and reusability. Biomass-derived cellulose nanofibers have been investigated as support materials because of their good thermal and chemical stability, wide surface area, and mechanical strength [28,29]. They have many hydroxyl groups, which make them efficient support materials for immobilized enzymes. Recently, 3D printing has been reported as being used to directly produce various support materials for enzyme immobilization [30], and 3D printing has the advantage of enabling the accurate manufacture of complex support structures in short periods without the use of harmful solvents [31,32]. 

Enzyme immobilization methods can be classified into three types: carrier binding, cross-linking (carrier-free binding), and entrapment. The amount of immobilized enzyme and the retained catalytic activity differ depending on the immobilization method and the properties of the enzyme, such as molecular weight, amino acid composition, and conformational structure. The advantages and disadvantages of each immobilization method are listed in Table 1. In this section, enzyme immobilization methods suitable for the degradation of pollutants in reactors are discussed. Their applications in degradation reactions using immobilized enzymes are described in Section 4. Recent enzyme immobilization methods for other applications in various fields, including industrial uses, have been described in a recent review [19,20,33].

### 2.1. Carrier Binding

**Figure 1:**
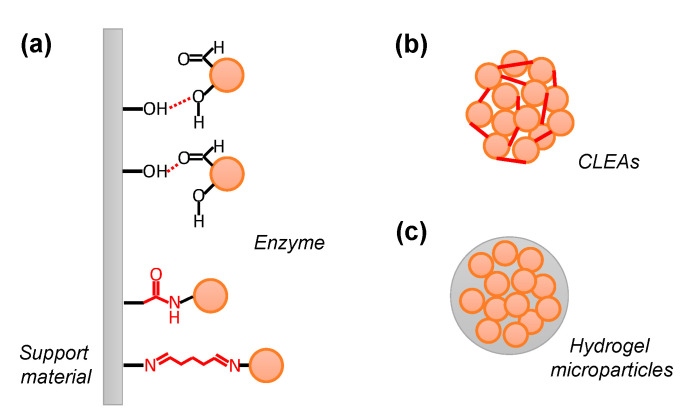
Enzyme immobilization methods. (**a**) Adsorption via hydrogen bonds or dipole-dipole interactions between enzyme polar surfaces and polar support materials. Covalent immobilization between enzyme residues and active support materials. (**b**) Enzyme polymerization via covalent bonds between enzymes. (**c**) Entrapment via physical interaction inside the hydrogel.

In the carrier-binding method, a water-soluble enzyme is immobilized on an insoluble support material through physical or chemical interactions, such as adsorptive, ionic, covalent, or affinity binding (Figure 1a). An optimal support material should be structurally stable and chemically inert and have a large solvent-accessible surface area. 

#### 2.1.1. Adsorptive Binding

Immobilization by adsorptive binding is an easy method involving surface interactions between the enzyme and the support material under suitable pH and ionic strength conditions [34]. Van der Waals interactions, hydrogen bonds, and dipole-dipole interactions are important for adsorptive binding. Immobilization by adsorption does not require chemical modification of enzymes and does not affect their conformational structure, with the expectation that enzyme activity will be retained after immobilization. However, since the interaction between the enzyme and its support material is weak, the enzyme may easily leak from the support material due to the temperature and coexisting substances, which is disadvantageous. The typical support materials include silica gel, porous glass, titanium dioxide, and mesoporous silica [34]. 

#### 2.1.2. Ionic Binding

Proteins can be either positively or negatively charged due to the nature of the side chains of exposed amino acid residues on their surfaces. Therefore, enzymes can be immobilized on the surface of ion-exchange resins via electrostatic interactions [35]. This has advantages such as decreased enzyme deactivation, simple implementation, the ability to immobilize them under mild conditions, and the reuse of support materials. However, as with adsorptive binding, the weak interaction between the enzyme and the support material can cause leakage of the enzymes from the support. Typical binding carriers include diethylaminoethyl (DEAE)–cellulose, DEAE–Sephadex and monoaminoethyl-*N*-aminoethyl (MANAE)–agarose [36,37]. 

#### 2.1.3. Covalent Binding 

This immobilization method involves the formation of a covalent bond between the functional groups of the enzymes, such as the amino group of the Lys residue and the reactive group of the support materials. Compared with adsorptive and ionic binding, covalent binding shows superior operational stability and reusability because enzyme leakage is often minimized by stable covalent bonds [38]. 3-Aminopropyl triethoxysilane (APTES) can be used to introduce amino groups onto the surface of carriers in the absence of amino groups on support materials [39]. Although covalent binding is strong enough to hold enzymes on the support material, their enzymatic activity may decrease owing to chemical modifications. Therefore, the concentration of each reagent is important for the preparation of immobilized enzymes [24]. Conventionally, the Lys residues of the enzyme and the surface amino groups on the support materials are bound using glutaraldehyde to immobilize them [39,40]. A combination of 1-ethyl-3-(3-dimethyl aminopropyl) carbodiimide (EDC) and *N*-hydroxysuccinimide (NHS) has also been used to form amide bonds between the Asp and/or Glu residue(s) of enzymes and the surface amino groups on the support materials [39,41]. 

#### 2.1.4. Affinity Binding 

This immobilization method binds the enzyme to the support material using interactions between biomolecules such as enzyme-coenzyme, antigen-antibody, or metal-chelation interactions [28,42]. A major feature of the affinity binding method is that it does not require using a purified enzyme, as the specific affinity between the carrier and the target enzyme enables selective binding of the target enzyme to the carrier simply by mixing the cell disruption solution and the carrier. The introduction of an affinity tag into the enzyme must be performed using an expression system or chemical modifications.

### 2.2. Cross-Linking

Similar to the covalent binding method described above, the cross-linking method (enzyme polymerization method) uses a chemical bond between a functional group of the enzyme and a compound that reacts with it (Figure 1b). Using a cross-linking reagent with two or more reactive functional groups in a single molecule, such as glutaraldehyde, enzymes can be cross-linked to form insoluble aggregates that retain their enzymatic activity. As with covalent binding to the support material, decreased enzymatic activity due to chemical modification of the enzyme can often occur. Therefore, it is necessary to consider the optimization of concentration conditions, such as the cross-linking reagent for the target enzyme [24]. 

Cross-linked enzyme aggregates (CLEAs) have recently been developed for carrier-free enzyme polymerization [21,43]. In a typical procedure for the preparation of CLEAs, CLEAs are generally prepared by aggregating an enzyme with the addition of a precipitant such as (NH_4_)_2_SO_4_ or *t*-butanol and then cross-linking with glutaraldehyde [44]. A cross-linking reaction occurs between Lys residues on the surfaces of neighboring enzyme molecules and glutaraldehyde via Schiff’s base reaction. This procedure is simple and can be widely applied. In addition, the prepared CLEAs exhibit good catalytic activity, high operational stability, and reusability [45]. These superior properties can significantly reduce the cost of enzymes, making their industrial applications economically feasible. Every enzyme has a unique amino acid sequence and a number of cross-linkable Lys residues. Therefore, the preparation of CLEAs should be optimized, as described in a recent review [25]. Because immobilized enzymes prepared by adsorptive, ionic, or covalent binding are immobilized at one or several points on the support materials, these immobilized enzymes have a high degree of freedom in their conformational structures and can only be reused a limited number of times, as they are easily desaturated and deactivated. To overcome this, complexes of enzymes and poly-Lys have been cross-linked and conjugated to a support material [46]. This method can immobilize the enzyme at high concentrations, thus improving catalyst efficiency. In addition, this poly-Lys immobilization method showed the formation of a cross-linked complex while retaining the conformational structure of the enzyme, making it less susceptible to denaturation by temperature, organic solvents, and pH extremes. It was also possible to prepare an immobilized enzyme that could be reused more than 50 times [46]. 

### 2.3. Entrapment

The entrapment method, which involves physically trapping enzymes without chemical modification on hydrophilic materials, such as hydrogels, can be applied to various enzymes [47]. Some entrapment structures include lattices, in which the enzyme is incorporated into the lattices of the polymerized hydrogel, and microcapsules, in which the enzyme is encapsulated within a semipermeable capsule (Figure 1c). The mass transfer efficiency of the substrates is affected by the pore size of the polymerized hydrogel. In addition to synthetic polymers such as polyacrylamide gels, naturally derived polymers such as agar, gelatin, and calcium alginate are generally used for hydrophilic support preparation. When using synthetic polymers such as acrylamide, care must be taken because monomer molecules may inactivate enzymes. In contrast, when agar or gelatin is used, heat is applied to dissolve them, making them suitable for heat-resistant enzymes. 

Alginate is widely used in the entrapment of enzymes because of its ability to rapidly form polymerized hydrogels with cations, such as Ca^2+^ [48,49]. In addition, the alginate microcapsules can be easily separated by simple filtration. However, because the enzyme is usually smaller than the pore size of the alginate hydrogel (~200 nm), it can leak during enzymatic reactions. To overcome this problem, CLEAs (sizes ranging from 1–10 μm) were entrapped in alginate hydrogels. Xu and Yang reported the entrapment of tyrosinase-CLEAs in alginate hydrogels for the degradation of phenolic compounds; the CLEAs in alginate hydrogels retained 100% enzymatic activity after six cycles [50]. 

Enzymes used for immobilization are generally purified from living organisms (microorganisms, plants, or animals). The purification of enzymes requires complicated procedures and time. One reported method to overcome these problems involves entrapping microorganisms expressing the target enzyme in alginate hydrogels [51,52]. This idea has advantages such as the ability to use the metabolic and coenzyme regeneration systems of the bacterium itself; however, since it is an organism, it needs to be kept alive during the degradation of hazardous pollutants; therefore, it poses challenges in terms of operational stability and reusability. 

## 3. Reactor Types Using Immobilized Enzymes

An advantage of immobilized enzymes is that they can freely form shapes. Most of the reported immobilized enzymes are in the form of particles, but they are used in various forms, such as membranes and tubules, depending on their purpose. Therefore, they are used in various reactors. Controlling several reaction conditions, such as temperature, pH, pressure, substrate concentration, and stirring speed in the vessel and keeping the reaction conditions constant allows immobilized enzymes to work efficiently [53]. Reaction vessels using conventional chemical catalysts use high temperatures and pressures to increase reaction efficiency, but enzyme-immobilized reactors do not require such conditions and are characterized by a high reaction rate. In addition, these reactors can be used repeatedly, reducing cost and labor demands.

### 3.1. Stirred Reactor

Stirred reactors use a mixer to facilitate the mixing of the immobilized enzymes and target pollutants. Batch reactions using agitators (shakers) are also classified under this type of reactor. After each reaction, the immobilized enzyme is recovered via centrifugation, the use of a membrane at the drain, or magnetic separation (if the support is made of a magnetic material). The impeller shape, size, and mixing speed affect the performance of this type of reactor [54]. This reactor type costs less than other reactors. Its advantage is that it is simple to use with a stirring system; however, since the product is not promptly removed from the reaction vessel, enzyme activity may be reduced due to product inhibition. Therefore, continuous flow-based reactors are also well-studied in bioremediation using immobilized enzymes. 

### 3.2. Fixed-Bed Reactor

Fixed-bed reactors are also known as packed-bed reactors. Cylindrical vessels are filled with immobilized enzymes, and the reactants flow downwards to react and yield products. This reactor type has advantages such as a high reactivity-to-volume ratio since they can be densely packed with immobilized enzymes, while product inhibition is unlikely to occur because the product is removed quickly. Moreover, they can be easily automated. Therefore, it is probably the most widely used reactor for the synthesis of intermediates and large-scale substances [33]. Recent reports have shown that enzymatic reactions in continuous-flow reactors can be more controlled, productive, and environmentally sustainable than other types [55]. To enable efficient reactions, the flow rate of the flow-based reactor should consider not only the shape and density of the immobilized enzyme but also the viscosity and density of the reactant. 

### 3.3. Fluidized-Bed Reactor

Fluidized-bed reactors are alternatives to fixed-bed reactors. In these reactors, the immobilized enzymes are suspended in cylindrical vessels and fluidized using a reactant flowing upward from the bottom. The flow rate is set considering the shape and density of the immobilized enzyme and the viscosity and density of the reactant, as in a fixed-bed reactor [53]. Fluidized-bed reactors have better mass transfer and heat performance than fixed-bed reactors and can efficiently degrade pollutants.

### 3.4. Enzymatic Membrane Reactor

In this method, the enzyme is immobilized on the surface of a polymeric membrane, such as a polyvinylidene fluoride (PVDF) membrane, via physical adsorption [56,57]. The substrate solution continuously separated the product by permeating the enzymatic membrane and enzyme inhibition by the products is unlikely to occur. 

## 4. Degradation of Pollutants Using Enzyme-immobilized Reactors

Bioremediation using enzymes can transform pollutants into less- or non-toxic forms. Laccases are frequently used for this purpose [58,59,60]. The use of laccase is strongly connected to its catalytic activity, which involves the oxidation of pollutants into radicals that can degrade into less toxic compounds than the initial pollutants [61]. Moreover, laccases catalyze the oxidation of a wide variety of organic compounds in the presence of oxygen without any additives, releasing water as the only byproduct. Therefore, it is an eco-friendly degradation enzyme. Among other laccases, fungal laccases such as those from *Trametes versicolor* have the highest potential for oxidizing phenolic compounds. The substrate specificity of laccase can be enhanced using redox mediators, not only phenolic compounds but also a wide range of other organic compounds [59]. Redox mediators are low-molecular-weight compounds that are easily oxidized by laccases and produce reactive radicals that can further oxidize complex organic compounds such as pollutants. In the laccase-mediator system (LMS) for degradation of pollutants, it was reported that 2,2′-azino-bis(3-ethylbenzothiazoline-6-sulfonic acid) (ABTS), 1-hydrobenzotriazole (HBT), or (2,2,6,6-tetramethylpiperidin-1-yl)oxyl (TEMPO) could enhance the oxidation rate of the laccase substrates [62,63]. Although these synthetic mediators are useful for the reactions of organic compounds, there are still challenges in their utilization in industrial processes owing to their high cost, toxicity, and reuse-associated problems. Recently, to overcome these challenges, low-cost and eco-friendly natural mediators, such as acetylacetone (AA) and acetosyringone (AS), have been used in LMS [64,65]. 

In this section, research progress on the use of enzyme-immobilized reactors for the degradation of pollutants (dyes, phenolic compounds, and pharmaceuticals) is discussed.

### 4.1. Dyes

Dyes are usually synthetic organic compounds that are widely used in many industries, such as textiles, food, paper production, and pharmaceuticals [66]. Currently, more than 40,000 dyes are used industrially. Owing to their intended use, these dyes must be highly durable and have a high color intensity. They are chemically and biologically stable and, hence, resistant to biodegradation. Furthermore, they can cause health problems [67,68], making it desirable to remove or degrade them from the environment. The properties of the enzyme-immobilized reactors used for dye degradation are summarized in Table 2. 

Many support materials have been used for enzyme immobilization via physical interactions. Wen et al. studied the performance of laccase immobilized on kaolinite for malachite green (MG) degradation [69]. Kaolinite is an aluminosilicate mineral that can be used as a support material for enzyme immobilization owing to its low cost, reusability, and structural stability. Kaolinite has negative sites on its surface; thus, laccase can be immobilized by adsorptive and ionic binding. The immobilized laccase exhibited good operational stability while maintaining 50% of its original enzymatic activity. With 3, 5-dimethoxy-4-hydroxybenzaldehyde (SA) as a mediator, the batch reactors with immobilized laccase showed MG degradation of nearly 80% at 30 °C in 300 min after five cycles. Recently, nanoparticles have been widely used as support materials because of their high surface-to-volume ratios [70]. Birhanlı et al. prepared Co- or Cu-based metal-organic frameworks (MOF), and laccase was immobilized on the nanoparticles via physical encapsulation [71]. The immobilized laccase exhibited good operational stability and maintained 55% enzymatic activity after 12 cycles. In the absence of any mediator, the batch reactors (12-well plates) with immobilized laccase into Co-based MOF degraded Reactive Blue 171 (RB171) and Reactive Blue 198 (RB198) by 78% and 61% at 50 °C in 60 min after five cycles, respectively. In another study, Yang et al. synthesized bimetallic Cu/Zn zeolitic imidazolate frameworks (ZIF) as support materials for laccase [72]. The immobilized laccase exhibited high operational stability in various interfering environments. In the absence of any mediator, the batch reactors with immobilized laccase degraded Reactive Deep Green KE-4BD (RG), Reactive Deep Blue B-2GLN (RB), and Acid Red 18 (CR) by 69%, 54%, and 45% in 240 min after five cycles, respectively. 

Nanoparticles are widely used, as described above. However, these materials are likely to exhibit cytotoxicity [73] and involve toxic organic solvents for chemical synthesis. To overcome these issues, bionanomaterials have been proposed as support materials [70]. Protein nanocages (vault nanoparticles) are one such bionanomaterial used for enzyme immobilization. Gao et al. prepared laccase immobilized on vault nanoparticles via physical adsorptive binding [74]. Human vault particles were prepared and purified using yeast. Results demonstrated improved degradation and detoxification of Reactive Blue 19 (RB19) and Acid Orange 7 (AO7) by immobilized laccase in batch reactors (Erlenmeyer flasks). Magnetic nanoparticles, which can be easily separated and recovered from magnetic materials using a magnetic field, have also been used as support materials. Li et al. prepared laccase immobilized on Fe_3_O_4_ magnetic nanoparticles for the degradation of dyes [75]. Carboxyl- and hydroxyl-modified magnetic nanoparticles were prepared and chelated with Cu^2+^ to immobilize laccase via metal-affinity binding. Laccase-immobilized magnetic nanoparticles are easy to handle and exhibit good operational stability. In the presence of ABTS as the mediator, the stirred reactors with immobilized laccase degraded MG, Brilliant Green (BG), Crystal Violet (CV), Azophloxine, Procion Red MX-5B, and RB19 at rates of 94%, 80%, 71%, 78%, 60%, and 65% at 50 °C in 150 min after 10 cycles, respectively. 

In another study, fixed-bed microfluidic reactors with laccase immobilized on magnetic nanoparticles were prepared for the degradation of Eriochrome Black T [76]. Laccase was covalently immobilized on the APTES-modified silanized magnetite nanoparticles using glutaraldehyde. The immobilized laccase showed a dye degradation of 93% without a mediator. The authors designed several microreactors based on torus geometries and discussed the potential applications of enzyme-immobilized microfluidic reactors for the treatment of wastewater rich in contaminant dyes in continuous flow systems. Svetozarević et al. reported another fixed-bed microfluidic reactor for dye degradation [77]. Peroxidase, another oxidase often used for bioremediation, has been crosslinked to form an enzymatic membrane on the inner polytetrafluoroethylene wall. The peroxidase-immobilized microreactor efficiently degraded Acid Violet 109 (AV 109) with high operational stability, retaining 65% of its initial activity after 10 cycles. CLEAs are carrier-free cross-linking methods, meaning that they are more cost-effective than other immobilization methods that use support materials. Hence, the ability of laccase-CLEAs to degrade dyes was tested [78]. Laccase-CLEAs showed good operational stability in the batch reactor (centrifuge tube), with their degradation activities being 89% and 12% at the sixth cycle for MG and RB2, respectively. These results suggest that low-cost enzyme immobilization methods, such as CLEAs, are cost-effective, eco-friendly, and have great scale-up potential. 

As discussed above, many immobilized enzymes have been prepared by various immobilization methods, and they show good operational stability, dye degradation efficiency, and cost-effectiveness. However, degradation systems that use immobilized enzymes often require expensive and toxic redox mediators. Immobilization allows the reuse (recovery) of enzymes but not redox mediators. To overcome this challenge, the co-immobilization of laccase and mediator systems has been reported. Sun et al. co-immobilized laccase and AA within a hydrogel (Immo-LMS hydrogel) and tested its performance in MG degradation [79]. The degradation ability of immobilized laccase without co-immobilized AA was reduced to 33% at the sixth cycle. In contrast, the degradation ability of Immo-LMS hydrogel remained above 87% at the seventh cycle, as expected. In a similar study, laccase-encapsulated Cu/ZIFs and ABTS were co-immobilized within polyvinyl alcohol hydrogels [80]. The co-immobilized laccase and ABTS could be reused to degrade MG multiple times, and their degradation ability remained above 69% after five cycles. These reports suggest that the co-immobilization of enzymes and mediators is a promising and cost-effective approach to reducing secondary pollution caused by mediators for industrial applications. 

**Table 2:** Degradation of dyes using enzyme-immobilized reactors.

Enzyme	Immobilization Method	Support Material	Reactor	Pollutant	Degradation ^a^	Conditions	Ref.
laccase	physical adsorption	kaolinite	Batch	MG	80% (10 mg/L)	0.5 mM SA, 30 °C, 300 min	[69]
laccase	physical encapsulation	Co/Cu-MOF	batch (12 wells plate)	RB171, RB198	78% (200 mg/L), 61% (150 mg/L)	50 °C, 60 min	[71]
laccase	physical encapsulation	Cu/Zn-ZIF	Batch	RG, RB, CR	69% (50 mg/L), 54% (50 mg/L), 45% (50 mg/L)	240 min	[72]
laccase	physical adsorption	vault nanoparticles	batch (flask)	RB19, AO7	72% (50 mg/L), 80% (50 mg/L)	27 °C, 8–24 h	[74]
laccase	affinity binding	Fe_3_O_4_@C-Cu^2+^	Batch	MG, BG, CV, Azophloxine, Procion Red, RB19	94% (50 mg/L), 80% (40 mg/L), 71% (5 mg/L), 78% (50 mg/L), 60% (20 mg/L), 65% (100 mg/L)	25 μM ABTS, 50 °C, 150 min	[75]
laccase	covalent binding	magnetite nanoparticles	microfluidic reactor	Eriochrome Black T	93% (20 mg/L)	12 mL/h for 25 min	[76]
peroxidase	cross-linking	− ^b^	microfluidic reactor	AV109	65% (10 mg/L)	0.2 mM H_2_O_2_	[77]
laccase	CLEAs	− ^b^	batch (tube)	MG, RB2	89% (500 ppm), 12% (100 ppm)	50 °C, 120 min	[78]
laccase	entrapment	CTS-g-PAM hydrogels	Batch	MG	87% (50 μM)	co-immobilized AA, 25 °C, 120 min	[79]
laccase	entrapment	PVA@Cu-ZIF hydrogels	Batch	MG	90% (10 mg/L)	co-immobilized ABTS, 55 °C, 300 min	[80]

^a^ Initial dye concentration in brackets. ^b^ Carrier-free immobilization.

### 4.2. Phenolic Compounds

Some phenolic compounds have carcinogenic or endocrine-disrupting effects. As laccase is a phenol oxidase, it can target compounds such as phenols and bisphenols for degradation without any mediators [81]. The properties of the enzyme-immobilized reactors used for the degradation of phenolic compounds are summarized in Table 3. 

Chlorophenols are used in pesticide manufacturing, wood preservers, intermediates in various pharmaceuticals, and dye formulations and are released as metabolites during the biodegradation of some pesticides. Some of these compounds have been classified as possibly carcinogenic to humans [82,83]. Among chlorophenols, pentachlorophenol (PCP) is a major health hazard to living organisms. Magnetic porous laccase CLEAs (Mp-CLEAs) have been synthesized for PCP degradation [84]. In a stirred reactor containing Mp-CLEAs, the degradation of 100 ppm of PCP was found to be 65% over 48 h at 50 °C. The authors also reported that the Mp-CLEA degradation system was improved by the addition of surfactants and mediators. The same authors reported another PCP degradation system using immobilized Lac. Here, superparamagnetic iron oxide nanoparticle-incorporated polymeric membranes were synthesized for laccase immobilization [85]. Immobilized laccase exhibited good operational stability. Using the immobilized laccase, 100 ppm of PCP was degraded by 62% over 24 h at 50 °C. These results suggest that immobilized laccase, with its improved mechanical and thermal stability, reusability, and storage, has good potential for degrading chlorophenols in wastewater. 

Bisphenol A (BPA) is widely used in the manufacture of polymers; however, it is an endocrine-disrupting chemical that is often detected in industrial wastewater. As BPA can interfere with the action of estrogen in vivo [86], an efficient degradation method is necessary. To this end, many studies using immobilized laccase or tyrosinase (another phenol oxidase) to degrade BPA have been conducted. Lin et al. immobilized laccase on Cu^2+^- and Mn^2+^-chelated Fe_3_O_4_ magnetic nanoparticles via metal affinity binding [87]. In a stirred reactor containing immobilized laccase, over 85% of BPA was removed under optimal conditions. Laccase has also been immobilized on amino-functionalized magnetic nanoparticles via CLEA formation [88]. The laccase CLEAs in the stirred reactors could degrade 87% of BPA (concentration of 60 ppm) over 11 h. Similarly, Taboada-Puig et al. used a combination of CLEAs containing peroxidase and glucose oxidase (combi-CLEAs) for BPA and nonylphenol degradation [89]. Within 10 min, combi-CLEAs degraded almost all phenolic compounds. In addition, a continuous membrane reactor with combi-CLEAs almost completely removed the BPA (10 mg/L) within 43 h. 

Enzymatic reactions in continuous-flow-based reactors can be more controlled, productive, and environmentally sustainable than other methods. Based on this idea, phenolic compounds have been degraded in a flow reactor using immobilized laccases prepared using various immobilization methods. Xia et al. immobilized laccase on polyethylenimine-modified Fe_3_O_4_ magnetic nanoparticles via covalent binding [90]. Efficient phenol degradation using immobilized laccase in a fixed-bed reactor has been reported, suggesting this is a promising method for the continuous degradation of phenolic compounds. Laccase immobilized on silanized Al_2_O_3_ nanoparticles and entrapped in alginate hydrogel microcapsules was prepared to study the degradation efficiency of acetaminophen, a phenolic pharmaceutical compound [91]. In a continuous-flow packed-bed microreactor, the entrapped laccase exhibited enhanced degradation efficiency. The phytotoxicity of water treated with the entrapped laccase was lower than that of untreated wastewater. In similar studies, laccase has been immobilized on polyacrylonitrile [92], in mesoporous silica [93], or on PEG-acrylamide resins [46]. Their degradation abilities against BPA and phenolic compounds have also been tested in continuous-flow reactors, with reports suggesting that degradation using immobilized laccase in a flow reactor system is an efficient and low-cost method for removing phenolic compounds from wastewater. 

**Table 3:** Degradation of phenolic compounds using enzyme-immobilized reactors.

Enzyme	Immobilization Method	Support Material	Reactor	Pollutant	Degradation ^a^	Conditions	Ref.
laccase	CLEAs	magnetic porous	Batch	PCP	65% (100 ppm)	0.1 mM 2,6-DMP, 50 °C, 48 h	[84]
laccase	cross-linking	PEES/PMVEAMA	Batch	PCP	62% (100 ppm)	0.1 mM 2,6-DMP, 50 °C, 24 h	[85]
laccase	affinity binding	Cu^2+^@Fe_3_O_4_	Batch	BPA	85% (20 mg/L)	30 °C, 12 h	[87]
laccase	CLEAs	Fe_3_O_4_	Batch	BPA	87% (60 ppm)	45 °C, 11 h	[88]
peroxidase, glucose oxidase	combi-CLEAs	C-Cu^2+^@Fe_3_O_4_	membrane reactor	BPA	100% (10 mg/L)	0.55 mL/min, 43 h	[89]
laccase	covalent binding	PEI-Fe_3_O_4_	fixed-bed reactor	phenol	70% (50 mg/L)	25 μL/min for 43h	[90]
laccase	entrapment	alginate@Al_2_O_3_	fixed-bed reactor	acetaminophen	72% (18 mg/L)	2 mL/h, 30 min	[91]
laccase	covalent binding	PAN	fixed-bed reactor	nonylphenol, octylphenol	60% (1 mM), 80% (1 mM)	25 °C, 90 min	[92]
laccase	cross-linking	porous silica	fluidized-bed reactor	BPA	80% (25 mg/L)	28.8 mL/min for 6 h	[93]
laccase	CLEAs	PEGA	fixed-bed reactor	BPA	2880 μM/h (100 μM)	50 °C	[46]

^a^ Initial phenolic compounds concentration in brackets.

### 4.3. Pharmaceuticals

Pharmaceuticals are indispensable in our lives. However, pharmaceuticals in wastewater can affect the environment and cause health problems in living organisms [94]. For example, anti-cancer drugs exhibit cytotoxicity and teratogenicity even at low concentrations [95,96]. As these compounds are not completely removed by currently used wastewater plants [97], they can be excreted into the environment as biologically active compounds or metabolites [98]. Therefore, in recent years, there has been growing interest in the removal and degradation of these compounds. Since 2020, research on the degradation of pharmaceuticals by laccases has increased [99,100,101]. The properties of recent laccase-immobilized reactors for the degradation of pharmaceuticals are summarized in Table 4. 

Naghdi et al. proposed oxygen-functionalized nanobiochars as support materials [102]. *T. versicolor* laccase immobilization was mediated by ionic binding between the carboxylic groups on the surface of the nanobiochars and the amino groups of laccases. The immobilized laccase prepared in a stirred reactor (50 mL flask) was tested for its degradation of carbamazepine (CBZ), an antiepileptic compound that has been frequently detected in wastewater. The degradation efficiency gradually decreased from 83% to 6% after seven cycles. The p*I* value of the *T. versicolor* laccase was 5.8, indicating that the Lys residues on the surface of the laccase were insufficient for ionic binding. Therefore, the interaction between laccase and nanobiochars was weak, and the loss of activity could be due to laccase leakage during the washing procedure. Recently, laccase was covalently immobilized on magnetically modified biochars via cross-linking [103], and the degradation of antibiotics (norfloxacin, enrofloxacin, and moxifloxacin) by the immobilized laccase was tested; better operational stabilities than those reported by Naghdi et al. [102] were shown, as expected. The removal efficiencies of immobilized laccase with ABTS mediator in the batch reactors (centrifuge tube) for norfloxacin, enrofloxacin, and moxifloxacin were 94%, 65%, and 77% at 40 °C after 48 h, respectively. In addition, the synergistic effect of the adsorption of antibiotics by biochars and their degradation by immobilized laccase resulted in high removal [103]. ZIF was used as another effective porous support material, and the synergistic adsorption and degradation of CBZ by laccase immobilized on ZIF were studied [104]. Laccase was stably and strongly incorporated into the ZIF particles. The immobilized laccase in the stirred reactors (50 mL tube) exhibited improved CBZ degradation efficiency (92%) and good operational stability. 

Membrane reactors immobilized with laccase, tyrosinase, and peroxidase have been studied for the degradation of several pharmaceutical compounds [105]. Each enzyme was immobilized on a cellulose membrane with a 5 kDa pore size. The results clearly show that the type of pollutant, as well as the type of enzyme, strongly affect the efficiency of enzymatic wastewater treatment. In another study, a PVDF membrane modified with multi-walled carbon nanotubes (MWCNTs) was used as support material for laccase immobilization [57]. Adding MWCNTs to a PVDF membrane not only improved the physical properties of the membrane but also enhanced the rate of electron transfer between the laccase and the substrate [106]. Laccase was covalently immobilized on PVDF/MWCNT membranes using the EDC/NHS method. The immobilized laccase was used to degrade CBZ and diclofenac. Degradation efficiencies of 95% at 4 h and 27% at 48 h were observed for diclofenac and CBZ, respectively, in the membrane reactor. The low degradation efficiency of CBZ compared to other studies may be due to the presence of an electron-absorbing amide group, which minimizes the electron transfer between CBZ and laccase on the PVDF/MWCNT membrane [57].

Efficient degradation systems using continuous-flow reactors have been developed. Poly Lys-supported laccase-CLEAs have been fabricated to degrade endocrine-disrupting chemicals [107]. Estrogens and anti-inflammatory drugs were tested as model endocrine-disrupting chemicals for laccase-CLEAs in a continuous-flow microreactor (fixed-bed reactor). The degradation efficiency of the system was better than that of other conventional reactors. Furthermore, it enables a two-step degradation in laccase-mediated reactions, thereby avoiding the inactivation of laccase-CLEAs. Another study reported on using immobilized laccase in continuous-flow reactors to degrade the anti-cancer drug tetracycline [108]. Laccase was immobilized on the gelatin beads via cross-linking. The degradation efficiency of immobilized laccase in a fluidic bed flow reactor is almost five times higher than that in a stirred reactor [108]. These reports indicate that using a continuous degradation system with fluid control is better than a conventional reactor, providing a simple catalytic system that can effectively remove pharmaceuticals. 

**Table 4:** Degradation of pharmaceuticals using enzyme-immobilized reactors.

Enzyme	Immobilization Method	Support Material	Reactor	Pollutant	Degradation ^a^	Conditions	Ref.
laccase	ionic binding	nanobiochar	batch (flask)	CBZ	83% (20 ng/L)	25 °C, 24 h	[102]
laccase	cross-linking	magnetic biochar	batch (tube)	norfloxacin, enrofloxacin, moxifloxacin	94% (10 mg/L), 65% (10 mg/L), 77% (10 mg/L)	1 mM ABTS, 40 °C, 48 h	[103]
laccase	physical encapsulation	ZIF	batch (tube)	CBZ	92% (5 mg/L)	24 h	[104]
laccase, tyrosinase, peroxidase	physical adsorption	cellulose membrane	membrane reactor	tetracycline	60% (1 μg/L), 50% (1 μg/L), 23% (1 μg/L)	5 mM ABTS, 25 °C, 24–40 min	[105]
laccase	covalent binding	PVDF/MWCNTs	membrane reactor	CBZ, DCF	95% (5 ppm), 27% (5 ppm)	25 °C, 4–48 h	[57]
laccase	CLEAs	− ^b^	fixed-bed reactor	E1, E2, EE2, NPX, DCF	>99% (18 μM), >99% (18 μM), >99% (18 μM), 71% (18 μM), 90% (18 μM)	0.5 mM ABTS, 0.5 μL/min	[107]
laccase	cross-linking	gelatin beads	fluidized-bed reactor	tetracycline	72% (20 ppm)	15 mL/min, 25 °C	[108]

^a^ Initial pharmaceuticals concentration in brackets. ^b^ Carrier-free immobilization.

## 5. Conclusions and Future Perspective

In this review, the recent advancements in enzyme-immobilized reactors for the degradation of hazardous pollutants were discussed. In addition to the papers presented in this review, a number of other studies have reported the application of immobilized enzymes in bioremediation. These results indicated an improvement in the operational and thermal stability of the immobilized enzyme compared to that of the free enzyme. In addition, efficient degradation and removal methods using immobilized oxidases (especially laccases) have been proposed for the detection of harmful pollutants with various chemical structures. As shown in Table 2, Table 3 and Table 4, the immobilization method and type of support material did not significantly affect the compound degradation. It is expected that various reaction conditions (high temperature, high pressure, and/or pH other than neutral) can be used, depending on the purpose. 

Microplastics and nanoplastics are nonbiodegradable polymers. Recently, their contamination has become a critical ecological concern owing to their persistent presence in every aspect of the ecosystem and their potentially harmful effects. Therefore, it is important to use organisms and their enzymes for the biodegradation of such polymers [109]. Applied research on immobilized enzymes in this field is in its infancy [110], and future research results are of interest. 

Although biodegradation by enzyme-immobilized reactors shows good operational stability and efficient catalytic activity, further considerations, including cost, reusability, and throughput, remain to be addressed before bioremediation technology is applied in practice. At present, the cost of enzyme-immobilized reactors remains a great challenge to their translation in practical applications. Various support materials relying on the enzyme immobilization method have been developed; however, these are generally expensive and often use reagents that can be contaminants in their synthesis. The number of patents related to support materials for enzyme immobilization has been increasing year by year since 2010 [111]. New technologies, such as the fabrication process of magnetic supports for enzyme immobilization by 3D printing (US20210189374) registered in 2019 and the creation of hybrid magnetic support structures with water-insoluble polymers registered in 2017 (US20210275997), are expected to facilitate more practical use of immobilized enzymes in the industrial sectors. As discussed above, CLEA technology is a promising method of enzyme immobilization because it does not require a support material and only a small amount of reagent is used for the cross-linking reaction. Therefore, it may be more suitable for mass production at lower prices. Additionally, continuous-flow-based reactors containing CLEAs are being developed, which can be expected to realize efficient and high-throughput degradation by controlling the flow rate. Redox mediators are required for the efficient degradation and removal of various pollutants. In a continuous-flow-based reactor for biodegradation, these mediators must always be available for the enzymatic reaction. As described in Section 4, efforts have been made to establish co-immobilization methods for enzymes and mediators. Although they are still under development, the advent of realistically reusable reactors for biodegradation could reduce the cost of bioremediation technologies. Lifecycle assessment (LCA) and techno-economic analysis (TEA) are important when evaluating new technologies and systems. The environmental impact of the new technologies described above can be analyzed using LCA by taking into account energy consumption and other factors [18]. In addition, inexpensive mass-production methods of immobilized enzymes with high stability and reusability may improve cost-effectiveness and practical applications can be examined using TEA [18]. Overall, it is anticipated that the development of these novel and reliable techniques will facilitate the application of enzyme-immobilized reactors in bioremediation. 

## Figures and Tables

**Table 1:** The comparison of the enzyme immobilization method.

Method	Advantage	Disadvantage
Adsorptive binding	Simple preparation, less influence on the structure of the enzyme, low cost	Enzyme desorption
Ionic binding	Simple preparation, less influence on the structure of the enzyme, low cost	Enzyme desorption
Covalent binding	High stability	High cost, easy to affect the conformational structure of the enzyme
Affinity binding	Simple preparation	Chemical or genetic enzyme modification need
Cross-linking	Easy preparation, carrier-free, high immobilization stability	Cross-linking yield depends on the number of Lys on the protein surface
Entrapment	Easy preparation, low cost, high enzyme activity	Enzyme leakage

## Data Availability

Not applicable.
